# Information and communication technology, educational attainment, and disparity in health information from one’s personal social network: The J-SHINE 2017 cross-sectional study

**DOI:** 10.1371/journal.pone.0275285

**Published:** 2022-09-28

**Authors:** Takekazu Kitagishi, Daisuke Takagi

**Affiliations:** 1 Faculty of Medicine, The University of Tokyo, Bunkyo-ku, Tokyo, Japan; 2 Graduate School of Medicine, The University of Tokyo, Bunkyo-ku, Tokyo, Japan; University of Exeter, UNITED KINGDOM

## Abstract

Previous studies suggested that accessibility to others with useful health information depends on one’s educational background. While Information and Communication Technology is thought to affect health information disparities, it remains unclear whether it widens or narrows them. We aimed to examine how four types of communication media—face-to-face/telephone, E-mail, LINE, social network service (SNS)—modify the association between educational background and accessibility to useful health information in the personal network of Japanese adults. We used data from the third-wave Japanese Study on Stratification, Health, Income, and Neighborhood (J-SHINE) survey conducted in 2017, which targeted middle-aged adults living in four municipalities within Japanese metropolitan areas. The results demonstrated that SNS use moderated the gap between educational backgrounds, suggesting that SNS can be an interventional leverage to close a health-related information gap between socioeconomic positions.

## Introduction

Peers within one’s personal social network who provide useful health advice, knowledge, and suggestions help him/her access appropriate health-related information and acquire desirable health-promoting behaviors [1]. However, those with lower educational attainment are likely to have a smaller number of friends [2–6] and fewer informational support networks [7], suggesting the disparity between educational backgrounds in the availability of health information from one’s personal networks.

The development and dissemination of information and communication technology (ICT) have impacted this disparity by, for example, reducing the time and distance constraints contingent to the maintenance and building of social ties [8, 9]. Although the digital divide, or disparity, in ICT accessibility between socioeconomic statuses (e.g., income and education) in the early days of the Internet (around 2000) has been discussed in the literature [10–12], the spread of ICT in societies in the late 2000s significantly decreased the accessibility gap. In Japan, the Ministry of Internal Affairs and Communications [13, 14] reported that, in 2017, 94.8% of households had mobile devices (mobile phones, personal handy-phone systems, and smartphones) nationwide. The percentage of individuals owning smartphone increased from 14.6% in 2011 to 60.9% in 2017. The use of social networking services (SNS) also increased with the popularization of smartphones. A nationwide survey showed that the percentage of those who use any of SNS such as LINE, Facebook, and Twitter increased from 41.4% in 2012 to 71.2% in 2016 among Japanese citizens aged between 13 and 69 years old [15].

However, there have been two contradicting arguments on the influence of ICT with regard to the gap in availability of health informants in one’s personal network: one suggests that the disparity is widened by the dissemination of ICT in society, while the other suggests the narrowing of the disparity.

The “widening theory” highlights the disparities related to not only the accessibility to ICT but also how to use it [16]. For example, Cotten and Gupta [17] found that those with a higher level of education are more likely to utilize ICT to research health information than are those with a lower level of education. In addition, Lampe et al. [18] revealed that users are more likely to utilize SNS to connect with people with whom they already connect offline and less likely to utilize it to initiate new connections, suggesting that the characteristics of one’s offline social networks are reflected in online social networks. This suggests that the gaps in availability of health information in one’s offline personal network may not be filled by SNS use. Furthermore, some studies suggest that people’s social media networking is based on the homophily principle, that is, social media users are likely to form selective relationships [19, 20]. People who are interested in health may thus tend to select friends who are familiar with health information online, resulting in the widening of the health information disparity among the population.

On the other hand, the “narrowing theory” argues that ICT reduces the time and distance constraints of maintaining and building social ties [8, 9] and may reduce the disadvantages of those with these constraints when they connect with peers offline. In addition, while cognitive [21] and economic resources [22] associated with the maintenance of social ties differ by socioeconomic background, ICT compensates for the gap between these resources. Previous research suggests that utilization of the Internet helps disseminate health information especially to low-education and low-income groups, thereby reducing inequality regarding health information and access to medical care [23, 24]. Additionally, existing literature reveals that interest-based homophily is not a strong factor influencing network formation on social media [25], suggesting that people who are not interested in health can be exposed to others who are familiar to health information online.

Although findings from previous studies are inconsistent regarding whether ICT widens or narrows the disparity in the availability of informational networks for health between educational backgrounds, the types of communication tools used may determine the outcome. For example, while e-mail is mainly used for communicating with intimate and homogeneous peers [26, 27], they inhibit the building of new relationships with heterogeneous peers [28]. Within a network of homogeneous people, shared information tends to be similar and redundant, making obtaining new and useful information difficult [29]. Thus, e-mail communication may not bridge the gap between educational backgrounds in accessibility to peers with useful health information. LINE, which is the most used social media platform in Japan [14], is considered to have a similar effect as e-mail. It has a text-based communication function similar to mobile e-mail and text messaging. It differs from mobile e-mail in that three or more people can communicate in real-time—much like a chat application—and users can send messages to multiple members of an existing group simultaneously like a mailing list. Although there are few studies of social networks on LINE, it is believed to be often used to communicate with peers with whom one frequently interacts offline, and it may not be a medium for connecting with diverse and useful information sources, as conventional mobile e-mails [30]. Meanwhile, Steinfield et al. [31] and Hampton et al. [32] suggest that, on SNS platforms such as Facebook and Twitter, connections with diverse and heterogeneous people are likely to be established and maintained; thus, these platforms can serve as a medium for communicating useful information sources. Although a study argues that SNS is used as a communication tool for people who already know each other offline [18], given that it is an asynchronous communication tool for an unspecified number of people [33], SNS also provides more opportunities to connect with others with whom one cannot connect offline [32] compared to e-mail and LINE. Therefore, the use of SNS may bridge the gap between educational levels.

Based on these discussions, this study examined the following three hypotheses:

H1. Peers with whom one connects on SNS (Facebook, Twitter) are more likely to be useful health information sources than are others.

H2. The association between SNS (Facebook and Twitter) use and the usefulness of peers’ health information is stronger among the lower-educated group than among the higher-educated group.

H3. The differences between educational levels are not observed in the other media (face-to-face, telephone, e-mail, LINE).

If the above hypotheses are supported, it suggests that the dissemination of ICT, especially SNS, contributes to reducing the disparity between educational levels with regard to health information sources in one’s personal social network. It also implies that ICT can be used as interventional leverage to close a health-related information gap between socioeconomic positions.

## Methods

### Data

We used data from the third-wave survey of the Japanese Study on Stratification, Health, Income and Neighborhood (J-SHINE) performed in 2017. The first-wave survey was conducted in 2010 in four municipalities within the greater metropolitan areas of Japan, with a probabilistic sample of community-dwelling men and women aged 25–50 years, the details of which have been described elsewhere [34]. Briefly, respondents answered self-administered questionnaires using a computer-aided personal instrument. In the first survey, of the 13,920 subjects, 8,408 were contactable, of whom 4,357 participated. Of the 4,294 eligible participants, 2,961 also participated in the second-wave survey in 2012. In 2017, the third-wave survey was conducted for participants in the first- and/or second-wave surveys. They received a paper-based self-administered questionnaire from a trained surveyor at their home and returned it by mail. Of the 3,727 eligible subjects, 2,787 participated (response rate: 74.8%).

All participants provided written informed consent prior to participation. The study protocol and the informed consent procedure were approved by the Ethics Committee of the Graduate School of Medicine of the University of Tokyo (No. 3073-(1)).

### Personal social network

We used the name generator methods [35] to determine participants’ personal social network. They were asked to nominate four peers aged ≥20 years with whom they usually interacted, other than family members and relatives. We then asked respondents about the social and health-related behavioral characteristics of each peer.

The unit of analysis was thus each respondent-peer pair. Because the respondents nominated up to four peers, the number of observations included in the analyses was 9,851 respondent-peer pairs nested in 2,693 respondents after excluding pairs with missing information on outcome and explanatory variables (n = 921).

### Measurements

#### Outcome

Our outcome variable was usefulness of each peer as a health-related information source, as assessed by the respondents. To determine this, we posed the following question to the respondents: “Is the person well-informed about health-related information (healthy food, exercise, habits, knowledge related to medicine and health, etc.)?” Responses were provided based on five predetermined categories (“knows very much,” “knows more than a certain degree,” “knows a little,” “does not know much,” and “I [respondent] am not sure”). Respondents were also asked “How useful is the information from the person (not limited to health)?” The four possible responses were: “very useful,” “somewhat useful,” “not very useful,” and “I have never obtained information from him/her.” If the responses were “knows very well” or “knows more than a certain degree” to the first question and “very useful” to the third question, these peers were defined as a “useful health information source” and coded as 1; the others were coded as 0.

#### Explanatory variables

Our explanatory variables were respondents’ communication medium (face-to-face/telephone, e-mail [via mobile phone, smartphone, or PC], LINE, and SNS [Facebook or Twitter]) combined with each peer and the educational attainment of the respondents.

With regard to the face-to-face/telephone medium, we asked the respondents: “How often do you meet and talk with the person or talk on the phone (including mobile phones)?” The three predetermined answer categories were: “more than once a week,” “several times a month,” and “less than once a month.” Responses of “more than once a week” or “several times a month” were coded as 1; the others were coded as 0.

With regard to e-mail, LINE, and SNS, we asked the respondents: “How often do you communicate with the person on e-mail (via mobile phone, smartphone, or PC)/LINE/SNS (Facebook or Twitter)?” For each medium, the respondents marked one of four categories: “more than once a week,” “several times a month,” “less than once a month,” and “I do not use it with him/her.” Responses of “more than once a week,” “several times a month,” or “less than once a month” were coded as 1; the others were coded as 0.

Regarding the educational backgrounds of respondents, educational attainment of high school graduation or less was coded as 1 while that of college graduation or a higher educational qualification was coded as 0.

In addition, to examine whether the associations between the communication media and the peers’ usefulness in health information were modified by respondents’ educational backgrounds, we included the interaction terms of respondents’ educational attainment and the use of each communication medium as explanatory variables in the analyses.

#### Covariates

Control variables included respondents’ sex, age, equivalent income, marital status, employment status, regular SNS use for health-related information seeking, and health literacy. To differentiate between the sexes, men were coded as 0 and women as 1. Age was used as a continuous variable. We divided equivalent income into tertiles, and those with missing information on this variable were categorized into a fourth category as “missing.” Regarding marital status, those who had a spouse/partner were coded as 1, while others were coded as 0. Employment status fell under four categories: “full-time worker,” “part-timer,” “unemployed,” and “missing.” Regarding regular SNS usage, the respondents mentioned the frequency of reading posts about health-related information on SNS by choosing one of the following categories: “non-use,” “do not use very much,” “use occasionally,” and “use often.” Those who chose “use often,” “use occasionally,” and “do not use very much” were coded as 1, while others were coded as 0. Health literacy was assessed by posing the following three items derived from the Japanese version of the Health Literacy Scale by Ishikawa, Takeuchi, and Yano [36]: “You can choose information you need from a lot of information,” “You can judge how reliable the information is,” and “You can explain to others the information you obtain.” Responses were rated based on a 4-point Likert scale ranging from 1 (never) to 4 (often). The Cronbach’s α coefficient of these three items was 0.86, indicating good internal consistency. The simple arithmetic mean of the three items was used as a continuous variable in the analysis.

Additionally, peers’ sex, duration of relationship with the respondent, age, educational attainment, and employment status were also used as control variables. To determine the duration of relationship with the respondent, we asked the respondents to state the number of years since they met each peer and divided them into tertiles; peers with missing information on this variable were categorized into a fourth category as “missing.” Regarding peers’ age, we asked respondents if the peer is “almost the same age (within ±5 years old),” “younger than 5 years old,” or “older than 5 years old.” Peers whose educational attainment was high school graduation or lower were coded as 1; others were coded as 0. Peers’ employment status was classified into four categories: “full-time worker,” “part-timer,” “unemployed,” and “missing.”

### Statistical analysis

We used logistic regression models to analyze the results. In Model 1, we set the usefulness of peers as a health information source as an outcome, regressed on the use of each communication medium and the respondents’ educational attainment, adjusting for the covariates. In Model 2, we added the interaction terms of each communication medium and the respondents’ educational attainment to Model 1.

The unit of analysis was each respondent–peer pair. Robust error estimation was used to account for clusters by respondents [37]. The software used was Stata 15.1 (Stata Corp, Texas, USA).

## Results

Among the respondents, the percentage of females (56.6%) was higher than that of male (43.4%) (Table 1); 25% of respondents were high school graduates or had a lower educational qualification, which was comparable to those of the same generation living in the same areas as reported in the 2010 census conducted (28%). With regard to regular SNS use for health-related information seeking, 43.4% of respondents fell under the “use” categorization.

**Table 1:** Descriptive statistics.

Respondents (n = 2,693)			Peers (n = 9,851)		
	n	%		n	%
**Sex**			**Sex**		
Male	1169	43.4	Male	4233	43.0
Female	1524	56.6	Female	5594	56.8
**Educational background**			Missing	24	0.2
Junior college/vocational school or higher	2005	74.5	**The duration of the relationship**		
			Short	3355	34.1
High school or lower	688	25.6	Middle	3345	34.0
**Marital status**			Long	3083	31.3
Unmarried or separated	583	21.7	Missing	68	0.7
Married	2110	78.4	**Age**		
**Employment status**			Almost the same as the respondent	5257	53.4
Regular	1353	50.2	Older than respondent	2523	25.6
Non-regular	956	35.5	Younger than respondent	1991	20.2
Unemployed	327	12.1	Missing	80	0.8
Missing	57	2.1	**Educational background**		
**Equivalent income**			Junior college/vocational school or higher	2250	22.8
Low	817	30.3			
Middle	824	30.6	High school or lower	5868	59.6
High	678	25.2	Missing	1733	17.6
Missing	374	13.9	**Employment status**		
**Usual use of SNS**			Regular	5497	55.8
Non-use	1414	52.5	Non-regular	2978	30.2
Use	1169	43.4	Unemployed	1187	12.1
Missing	110	4.1	Missing	189	1.9
	**Mean**	**SD**	**Communication media**		
Age	45.5	7.02	Face-to-face/telephone		
Health literacy (range: 1–4)	1.75	0.67	Use	7565	76.8
			Non-use	2286	23.2
			E-mail (mobile/smartphone/PC)		
			Use	7327	74.4
			Non-use	2524	25.6
			LINE		
			Use	5837	59.3
			Non-use	4014	40.8
			SNS		
			Use	1096	11.1
			Non-use	8755	88.9
			**Health-related information**		
			Recognized as a useful health information source	1454	14.8
		
			Other	8397	85.2

Among peers, the percentage of females was 56.8%, which was comparable to the gender ratio of respondents. With regard to communication media, respondent–peer pairs communicating via face-to-face/telephone, e-mail, LINE, and SNS accounted for 76.8%, 74.4%, 59.3%, and 11.1%, respectively; 14% of peers were recognized as “useful health information sources” by respondents.

Model 1 in Table 2 shows that respondents’ lower educational background was negatively associated with the usefulness of peers as a health information source at the statistical significance level of .10 (odds ratio = 0.85; 95% confidence interval [CI] = 0.69, 1.05). The odds ratios of “face-to-face/telephone use,” “e-mail use,” “LINE use,” and “SNS use” were 1.18 (95% CI: 0.99, 1.41), 1.12 (95% CI: 0.94, 1.36), 1.05 (95% CI: 0.89, 1.24), and 1.27 (95% CI: 0.99, 1.63), respectively.

**Table 2:** Estimated results of logistic regression analysis using the usefulness of peer as a health information source as the objective variable.

	Model 1^a^	Model 2^a^
	OR^b^ (95% CI^c^)	OR^b^ (95% CI^c^)
**Educational background**		
Junior college/vocational school or more	Reference	Reference
High school or less	0.85 (0.69, 1.05)	0.92 (0.55, 1.55)
**Face-to-face/telephone**		
Use	1.18 (0.99, 1.41)	1.14 (0.94, 1.39)
Non-use	Reference	Reference
**E-mail (mobile/smartphone/PC)**		
Use	1.12 (0.94, 1.36)	1.25 (1.01, 1.54)
Non-use	Reference	Reference
**LINE**		
Use	1.05 (0.89, 1.24)	1.04 (0.86, 1.26)
Non-use	Reference	Reference
**SNS**		
Use	1.27 (0.99, 1.63)	1.15 (0.87, 1.53)
Non-use	Reference	Reference
**Face-to-face/telephone×Education**		1.23 (0.81, 1.86)
**E-mail use×Education**		0.61 (0.40, 0.94)
**LINE use×Education**		1.08 (0.73, 1.60)
**SNS use×Education**		1.63 (0.95, 2.83)

^a^ Odds ratios were adjusted for respondents’ sex, age, marital status, employment status, equivalent income, usual usage of SNS, health literacy, peers’ sex, duration of the relationship with the respondents, age, educational background, and employment status.

^b^ OR: odds ratio

^c^ CI: confidence interval

Model 2 in Table 2 shows the interaction terms between the use of each communication medium and respondents’ educational attainment (high school graduation or less). The odds ratios of “face-to-face/telephone × education,” “e-mail use × education,” “LINE use × education,” and “SNS use × education” were 1.23 (95% CI: 0.81, 1.86), 0.61 (95% CI: 0.40, 0.94), 1.08 (95% CI: 0.73, 1.60), 1.63 (95% CI: 0.95, 2.83), respectively.

**Fig 1:**
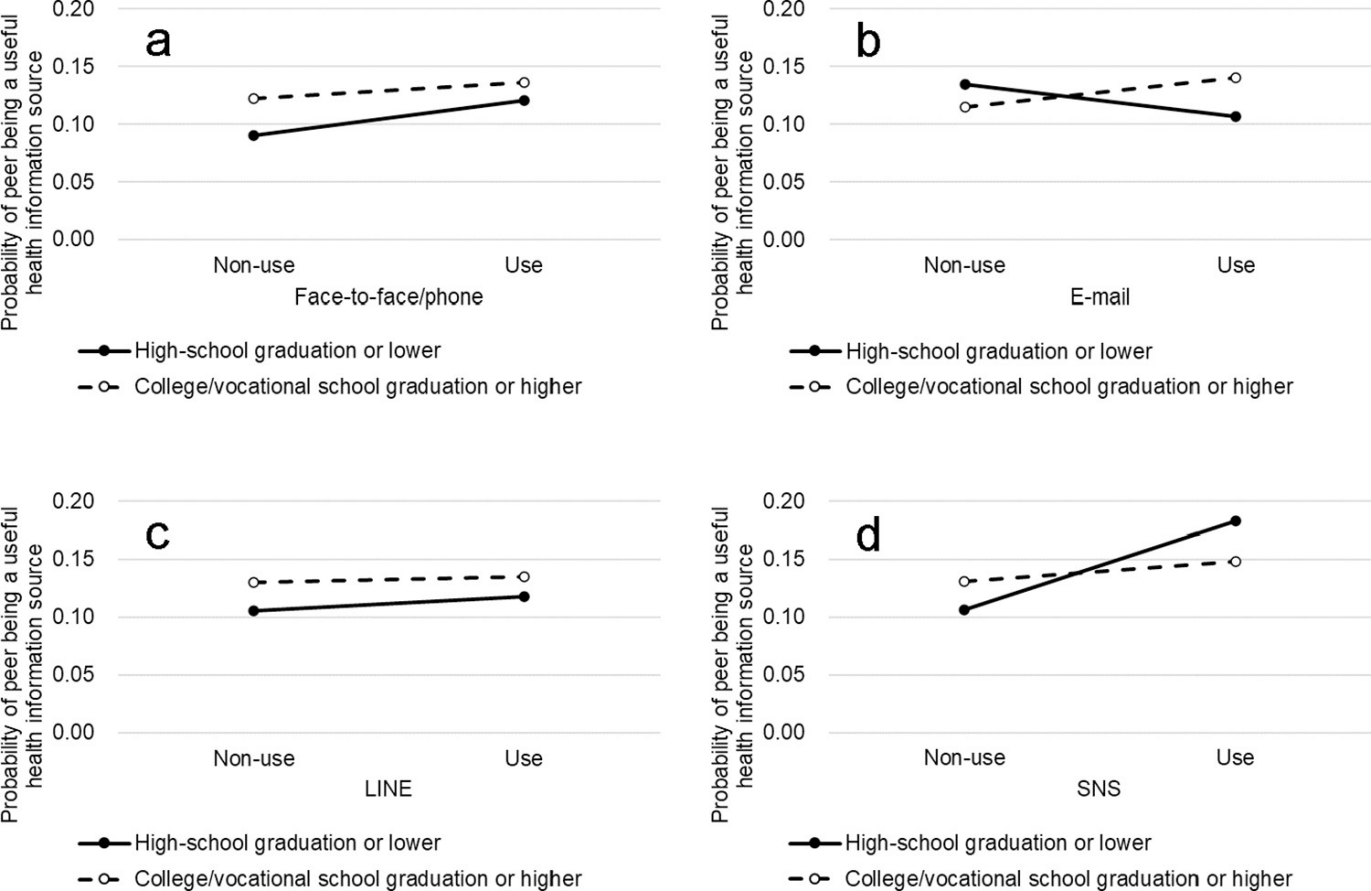
Predicted value of the probability that peers connected with each communication medium are useful information sources. a: Face-to-face/telephone; b: E-mail (mobile/smartphone/PC); c: LINE; d: SNS.

Fig 1 shows the predicted probability of peers being assessed as useful health information sources by the types of communication media and respondents’ educational attainment, which were calculated based on the estimates for Model 2 in Table 2. Other variables were set as their mean values. Panel d reveals that the association between SNS use and peers’ usefulness was stronger among those with lower education than among those with higher education. Panels a and c reveal that the associations of face-to-face/telephone use and LINE use with peers’ usefulness were only slightly stronger among respondents with lower educational attainment. Although e-mail use was positively associated with peers’ usefulness among highly educated respondents, it showed a negative association among respondents with lower educational attainment (Panel b).

## Discussion

In this study, the percentage of respondents who used SNS was 43.4% (Table 1), which was lower than the SNS usage rate of 61.1% among the 40–49 age group reported by a representative national survey for Japan conducted in 2017 [38]. As the present study asked the respondents about their SNS usage limited to the purpose of health information seeking, their general experience of using SNS was likely to have been underestimated. Additionally, as the Ministry of Internal Affairs and Communications reported the proportion of SNS users only among Internet users [38], it may be overestimated compared to that of the present study. Therefore, the familiarity of this study’s respondents with SNS did not seem to deviate significantly from that of the Japanese population as a whole.

Additionally, among our respondents, the proportions of those who had used the Internet, including websites other than SNS, to seek health information were 90.1% for those with high school graduation or lower qualifications and 93.3% for those with college graduation or higher qualifications (data not shown). A previous study conducted in the U.K. reported that the proportions of those who used the Internet to seek health information in 2019 were 73.1% for those with lower educational attainment and 83.1% for those with higher educational attainment [39] (calculated from Estacio et al.’s [39] Table 1). Although the data was from 2011–12, a study in the U.S. also showed a large disparity between educational levels in Internet utilization for health information seeking (35.7% for those who “did not graduate from high school,” 57.3% for those with high school certification, 73.4% for those with a college degree, and 81.2% for those with a graduate degree) [40]. Compared to these previous studies, in the present study the disparity between educational backgrounds in accessibility to the Internet for health information seeking was relatively small. Therefore, the question was whether the association between connecting with peers via SNS and the perceived usefulness of their health information differed by educational background.

Although previous studies suggested that those with lower educational attainment are less likely to connect with peers who are useful health information sources, how the disparity varies depending on the types of communication media used has been unclear. The present study adds novel findings to the existing knowledge by revealing that while the associations of lower educational background and SNS use with peers’ informational usefulness were negative and positive, respectively, peers connected via SNS were more likely to be useful information sources among respondents with lower education than among those with higher education.

Regarding the association between education and peers’ information usefulness, opportunities of social networking [2, 41], availability of expenditure for maintaining and building social ties [22, 42], and cognitive resources and skills [43, 44] that are determined by duration of education may contribute to the disparity in the number of peers with useful health information between educational levels.

The positive association between SNS use and peers’ information usefulness (Model 1 in Table 2) supports hypothesis 1. ICT reduces the time and distance constraints associated with maintaining and building social ties [8, 9]. Thus, even if peers with useful health information are far away or there is not enough time to meet them, relationships can still be easily maintained through SNS.

While peers with a high frequency of face-to-face/telephone interactions tended to be useful health information sources, this mode of communication was weaker than that of SNS (Model 1 in Table 2). Face-to-face/telephone interactions are more likely to be used to communicate with physically close peers [9] compared to SNS, and such peers share relatively high intimacy and would actively provide informational support. However, the information they provide may already be familiar to the respondent and may thus be assessed as relatively not useful.

Connecting with peers via e-mail was positively but weakly associated with their informational usefulness. Previous studies suggested that e-mail via mobile phones is likely to be used between longtime friends, contributing to form a homogeneous network [26, 27]. Information obtained from homogeneous peers is likely to be redundant and underestimated in terms of usefulness and novelty [29]. Meanwhile, an existing study suggests that the use of PCs for e-mail is positively associated with users’ network diversity [45]. This study, however, did not distinguish the tools used for e-mail communications, and the estimated association may be a mixture of homogenous and heterogeneous effects.

Connecting via LINE was not associated with the informational usefulness of peers. Although there are no empirical studies examining how LINE is used to interact with others, it would mainly serve as a means of communication with peers connected offline, which may not contribute to additional connections with useful informational ties.

The interaction effect between connecting via SNS and educational background revealed that the association between SNS use and informational usefulness of peers was strengthened among respondents with lower education (Table 2, Model 2), supporting hypothesis 2. As shown in Panel d in Fig 1, among those with lower education, accessibility to useful health informational caught up with or even overtook that of those with higher education, suggesting that SNS may compensate for the disparity between educational backgrounds in terms of time, cognitive, and economic resources [21, 22] to maintain useful social ties. This also suggests that at least with regard to health informational resources in one’s personal network, the “narrowing theory” rather than “widening theory” is likely as a social consequence of the widespread use of SNS.

Connecting via face-to-face/telephone and LINE did not show clear interaction effects with respondents’ education compared to that via SNS, which supports hypothesis 3. While the association between frequency of face-to-face/telephone communications and peers’ informational usefulness was slightly strong among respondents with lower education (Panel a in Fig 1), the modification effect was weaker than that for SNS. LINE revealed a nearly null interaction effect with respondents’ education. Considering that LINE is mainly a means of communicating with offline connections, it would not compensate for the disparity in accessibility to peers with useful information between educational backgrounds.

Peers communicating via e-mail tended to be useful sources of health information only among highly educated respondents, suggesting a widening of disparity between educational levels (Panel b in Fig 1). Among the lower educated respondents, health information from peers interacting via e-mail was assessed as not useful. Given that the effects of e-mail communication on homogeneity/heterogeneity of social ties differ depending on its usage (e.g., using mobile phone or PC [26, 45]), the difference in the effect of e-mail observed in this study may reflect the different usage of e-mail between educational levels.

While previous studies have demonstrated that social ties are more likely to be formed and maintained among homogeneous people, for example, in terms of place of residence and shared social media content [46, 47], in this study, peers connecting via SNS showed higher heterophily regarding gender, age, educational attainment, working status, place of residence, hobbies/interests, and values for health than those connecting via other media, especially among respondents with lower educational attainment (data not shown). We assumed that such heterogeneity was effective in facilitating respondents’ access to useful health information. However, the kind of information that was actually exchanged via SNS and perceived as useful in one’s intimate personal network should be further examined, namely, whether heterogeneous information was provided by peers connected via SNS or homogeneous information was shared that the respondents found useful.

To the best of our knowledge, this is the first study examining the relationship between ICT use and health information disparities with regard to educational backgrounds. One of the strengths of this research is that we collected detailed information on the intimate peers of respondents, such as communication media, attributes, and health information, using the name generator. Second, since the subjects were extracted by strict probabilistic sampling from the population, the generalizability of this study’s findings to the study population is strong.

Some limitations of our study should be noted. First, data on peers was measured based on respondents’ subjective perceptions, which may be affected by their cognitive bias. Second, the data in this study cannot be generalized to the entire Japanese population. Third, because this study employed a cross-sectional design, causality cannot be clarified. In addition, it is unclear whether the use of SNS contributes to the maintenance or to the building of useful information networks. Nevertheless, given that the name generator in this study measured intimate peers and that such intimate relationships are less likely to be initiated only online, we assumed that the association between SNS use and connection with useful peers was attributable to increased maintainability of social ties due to SNS use. Finally, this study examined a small number of intimate peers (up to four) in respondents’ personal networks, failing to investigate nonintimate ties that may provide useful health-related information. Considering that the strength of SNS is to maintain and build such nonintimate social ties, this study may have underestimated the effect of SNS.

In conclusion, peers who connected via SNS tended to be useful sources of health information. The tendency was stronger especially among those with lower education. Connecting via face-to-face/telephone and LINE showed the weaker modifying effects than that of SNS, while connecting via e-mail showed the opposite—disparity widening—effect.

ICT will be more popularized in the future. This study suggested that the widespread use of SNS will be able to reduce the disparity in health-related information sources in personal social network between educational backgrounds. Therefore, the effective utilization of ICT in a society may lead to desirable social consequences with regard to people’s informational network environments. However, we should focus on the continuous changes in people’s interactions with ICT. For example, more wearable devices, such as smartwatches, are becoming popular as new ICT devices [48]. Smartwatch users utilize them primarily for fitness and communication purposes [49], including immediate response to text messages, emails, and SNS [49]. Wearable devices can make communication even more immediate and ubiquitous. Two questions present scope for further research: Can this contribute to increasing access to more diverse information and heterogeneous people? Conversely, as communications via smartwatch are simplified and suitable for informal and intimate interactions, can this strengthen communications with close and homogenous people? At present, there is no evidence of a relationship between the new ICT devices and the homophily of social networking. The relationships between the development of ICT and changes in people’s social networks warrant further investigation.
